# Supplementary material for: Determinants of knowledge of pregnancy danger signs in Indonesia
Source: PLoS One. 2020 May 20;15(5):e0232550. doi: 10.1371/journal.pone.0232550 (PMC7239433; doi:10.1371/journal.pone.0232550)
Supplement: Attachment — Submitted filename: The Determinant of Knowledge of The Pregnancy Danger.docx [file pone.0232550.s001.docx]

Thank you for giving me the opportunity to review the manuscript entitled: "The determinant of knowledge of the pregnancy danger signs"

However, It is unfortunate that the journal did not provide me the article with line numbers track, that makes a bit difficult to give more details comments.

Please find below is my comments:

- An English Language editing will make the article more interesting to read as I found some words were not put in the correct way, e.g. page 3 – almost 2 times that of Cambodia… etc.
- The data source was from the 2017 IDHS data meaning the data was gathered could be a year before or more. A new information may exist.
- It is unclear the inclusion criteria for a woman childbearing age (15-49), as 15 -19 used as reference for some data, were any of those women aged 15 years old experience pregnancy?
- Were there any data regarding the MMR in pregnancy because the women had chronic disease?
- The discussion was not support with a strong evidence from previous studies that explain the knowledge of pregnancy danger signs have a significant correlation with MMR
- The background need to be added with some literature that explains the gap in the literature why is so important to do this research.
