# Supplementary material for: Determinants of knowledge of pregnancy danger signs in Indonesia
Source: PLoS One. 2020 May 20;15(5):e0232550. doi: 10.1371/journal.pone.0232550 (PMC7239433; doi:10.1371/journal.pone.0232550)
Supplement: Attachment — Submitted filename: Response to Reviewers.docx [file pone.0232550.s002.docx]

**Response to Reviewers**

1) An English Language editing will make the article more interesting to read as I found some words were not put in the correct way, e.g. page 3 – almost 2 times that of Cambodia… etc.

*Thank you for the advice. The revised manuscript was professionally edited.*

2) The data source was from the 2017 IDHS data meaning the data was gathered could be a year before or more. A new information may exist.

*The latest official release of MMR is not yet available by the Indonesian government.*

3) It is unclear the inclusion criteria for a woman childbearing age (15-49), as 15 -19 used as reference for some data, were any of those women aged 15 years old experience pregnancy?

*Based on the information in Table 2, there are 444 women aged 15-19 who experience pregnancy.*

4) Were there any data regarding the MMR in pregnancy because the women had chronic disease?

*No information is available for the data was mentioned.*

5) The discussion was not support with a strong evidence from previous studies that explain the knowledge of pregnancy danger signs have a significant correlation with MMR

*Globally, at least one woman dies every minute during pregnancy and childbirth [1]. Deaths due to pregnancy complications and vaginal birth can be easily prevented [2][3]. Prevention can be done by increasing knowledge of the pregnancy danger signs, which has a strong correlation with early detection of pregnancy risks. Women who know the pregnancy danger signs are at 6.657 times more likely than those who do not understand about early detection of pregnancy risks [4]. Knowledge of pregnancy danger signs has a strong correlation with antenatal care [3]. Women who can identify the pregnancy danger signs are 3.470 times more likely to participate in antenatal care [5]. It is an evidence that knowledge of pregnancy danger signs as well as Maternal Mortality Rate (MMR).*

6) The background need to be added with some literature that explains the gap in the literature why is so important to do this research.

*Globally, at least one woman dies every minute during pregnancy and childbirth [1]. Deaths due to pregnancy complications and vaginal birth can be easily prevented [2][3]. Prevention can be done by increasing knowledge of the pregnancy danger signs, which has a strong correlation with early detection of pregnancy risks. Women who know the pregnancy danger signs are at 6.657 times more likely than those who do not understand about early detection of pregnancy risks [4]. Knowledge of pregnancy danger signs has a strong correlation with antenatal care [3]. Women who can identify the pregnancy danger signs are 3.470 times more likely to participate in antenatal care [5]. It is an evidence that knowledge of pregnancy danger signs as well as Maternal Mortality Rate (MMR).*
